# Transactions of the Provincial Medical and Surgical Association

**Published:** 1846-10

**Authors:** 


### Art. III.

*Transactions of the Provincial Medical and Surgical Association.* New Series. Vol. II.—*London,* 1846. 8vo, pp. 285.

The contents of the present volume are the following:—1, The Retrospective Address in Medicine, by Edward Charlton, M.D.; 2, The Retrospective Address in Surgery, by Thomas P. Teale, Esq.; 3, On Grinders' Asthma, by C. F. Favell, M.D.; 4, On the Inverted Displacement of the Urinary Bladder, by J. G. Crosse, Esq.; 5, Report of the Reading Dispensary for the years 1841-4, by Charles Cowan, M.D.; 6, A Statistical Report of the Surgical In-patients of the Royal Berkshire Hospital from 1839 to 1845, by George May, Esq.

I. Our readers have had so much from ourselves in the form of Reports of the progress of medical science in its different branches, that they will excuse us for not giving any account of the two excellent addresses with which the volume opens. We must also content ourselves with referring to the original report of the Berkshire Hospital and Dispensary, which do great credit to the industry and talents of their authors. It is surprising, when we consider the great number of hospitals scattered over all our counties, that so few of their brethren have emulated Dr. Cowan and Mr. May in enriching the pages of these Transactions with similar documents.

II. Mr. Crosse's paper gives the history of a case of rare occurrence, and is important, as exhibiting the value of a careful examination and consequent just diagnosis, and the dreadful risk attending a mistake in this particular. We extract the more material points of the narrative:

"In the year 1829, a highly respected colleague of mine, since deceased, received under his care a healthy-looking female child, aged between two and three years, on account of a tumour, about the size and shape of a walnut, projecting visibly at the external *labia pudenda.* It was of a florid red colour, and somewhat granulated upon its surface, so as to resemble a large strawberry; and the surgeon entertained a notion that it was a vascular tumour, which might be removed by ligature, on which account he requested me to inspect it.

"After a slight examination, I expressed my doubts as to its being a vascular tumour, and dissuaded him from the hasty application of a ligature. I could not, however, immediately explain its nature, having no conception how such a tumour could be formed by the displacement of parts only, without any superadded morbid growth. Towards the posterior part of the tumour, and on its sacral aspect, there was an aperture, which was conjectured to be the entrance into the displaced urethra. A very small female catheter easily entered this aperture, and passed along a channel a little to the left side of the median line: urine distilled in drops through the catheter, but there was not a gush, although the instrument had entered so far that we concluded it must have reached the cavity of the bladder. Besides what thus oozed through the catheter, slightly tinged with blood, there was an oozing of urine from another source, which was not explained until a second and more strict examination, instituted a few days afterwards, on my casually coming to the patient's bedside, just as the surgeon was prepared to apply a ligature round the neck of the tumour.

" I now found, concealed in a fold of the tumour, and near to the posterior unction of the labia, two orifices not far asunder, from which the urine oozed, and which were evidently the vesical terminations of the ureters. On pressing the tumour firmly, as if to reduce it like a hernia, I found it yield and pass gradually behind the symphysis pubis, and within the labia; and under a continuance of the taxis it all retired, leaving the external parts in their proper shape and position. A passage remained, through which the tumour on retiring had taken its course, which was actually the dilated urethra, into which I *could* and *did* introduce my little finger, until it fairly entered the cavity of the replaced bladder; for it now became clearly demonstrated that the vascular red tumour, externally presenting itself as first described, was the urinary bladder in its entire thickness, including its mucous, muscular, and peritoneal coats, prolapsed through the dilated urethra, and at the same time inverted or turned inside out. The proper lining membrane of the bladder became, in the progress of this displacement, the external covering of the tumour. As fast as the urine was secreted by the kidneys, it oozed from the terminating orifices of the ureters, which were concealed within a fold of the exposed surface of the tumour, and approximated to each other. The neck, or deepest and narrowest part of the tumour, just concealed within the labia, was covered by the inverted lining of the urethra, the inversion being complete.

" In this instance, had a ligature been efficiently applied to the neck of the tumour, as was contemplated, the bladder would have been removed, including all its coverings, the ureters cut through just above their terminating orifices, and the peritoneal cavity largely opened, with a necessarily fatal result!

" As the friends of the child could not be applied to, the history was imperfect. It was stated that the tumour had existed for a considerable time, and been always attended by stillicidium urinæ; also that it had been once replaced, but descended again, shortly before it came under my observation. During the short period that the child remained under my notice, after the replacement of the bladder, thare was no relapse; and since this account was sent to press, I have been fortunate enough to ascertain, and to be enabled to add, that the patient is still living, after an interval of sixteen years, and is a healthy young woman, save only the affliction of the incontinence of urine, with which she has been constantly troubled, but without any relapse of the vesical displacement.

III. We had occasion, some two years past,* to draw the attention of our readers to the disease familiarly known as Grinders' Rot or Asthma, in connexion with the work of Dr. Calvert Holland. The tolerably full notice we bestowed upon that work will save us from the necessity, in the present place, of entering into any preliminary details concerning the various modes of occupation and pursuit of the artisans who fall victims to this singular affection—singular, perhaps, rather in its peculiar mode of manifestation, than in the fact that some disease or other of the pulmonary organs should be the result of the mode of working pursued in Sheffield and its vicinity. Besides, much fuller (and, as far as may be judged from internal evidence, more correct) information on this branch of the subject may be derived from Dr. Holland's volume than from the essay now before us.

" The object which I have peculiarly in view, in the present communication," says Dr. Favell, " is to determine the pathology of grinders' asthma, or rather, perhaps, I should say, to exhibit the lesions of the respiratory organs, which morbid anatomy most frequently reveals in the persons of those who have fallen victims to this disease."

Here two things most different—the pathology and the morbid ana-

* Brit. and For. Med. Rev., October, 1844.

tomy of a disease—are spoken of as though they were all but one and the same. We notice this as a mere verbal inaccuracy. The author knows, as well as we do, the difference between them.

Dr. Favell says that the most remarkable physical signs observed in these cases are "more or less imperfectness of expansion in some portion of the chest, an abnormal amount of dullness on percussion, the substitution of the tubular for the vesicular murmur, and the occasional existence of amphoric resonance, cavernous respiration, and pectoriloquy." Now Dr. Holland, on his side, puts forth the following statements:—"The chest generally sounds well on percussion," "far better than would be anticipated from the pulmonary affection;" and, further on, the sound on percussion is described as "being frequently much louder than in health." The respiration is, according to the same writer, natural, puerile, or bronchial. Doctors will differ; so that there is nothing more than an amiable deference here to the common opinion, which hath made itself known in the well-worn saw. But if the cause of the difference be sought for, we are, we confess, puzzled. Can any of the "grit," or the "dust," or the "flying particles," so common in the locality, have obtruded itself into the ears of either observer, and transformed the sounds actually evolved into others harmonizing more or less distinctly with the theories held by each? But whatever be the explanation, the fact is a most singular one.

"Sensual gratification is the great object the grinders have in view,"—a character which is in nowise peculiar (as Dr. Favell's inference would seem to be) to the class of whose pulmonary diseases, he has composed a history. Nor do we, as a general fact, descry the utility or philosophy of seeking to enrol debauchery, venereal or spirituous, among the "causes" of the grinders' rot, when it is clear as the light of heaven, that if the poor wretches led the chaste and temperate lives of very anchorites, and continued to inhale this pernicious grit, they would perish victims of the "rot" its inhalation engenders. We have no doubt that spirituous drinks and consequent inebriety do greatly aggravate the local malady of these miserable men; but, at the same time, we say that it might be asserted with almost as fair a show of probability, and for anything shown to the contrary by the historians of the "rot," that habits of inebriety act rather in the direction of slackening than accelerating the speed with which the pulmonary disease runs its fatal course. This may appear a paradox to some pathologists (we mean those of the *à priori* school of reasoning),—but we profess to *observe*, and not to frame opinions for one sect or the other.

Dr. Favell relates some cases of the disease with the particular view of illustrating its morbid anatomy. From these cases it appears that the main morbid changes discovered are—"1, tubercles; 2, small bodies resembling currants disseminated extensively on the surface and throughout the substance of the lungs; 3, large masses found in different portions of the pulmonary tissue; 4, emphysema; 5, dilatation of the bronchial tubes; 6, inflammation of the lining membrane of the bronchi, trachea, and larynx; 7, adhesion of the pleuræ; 8, enlargement of the bronchial glands; 9, enlargement of the heart; 10, a granular condition of the kidneys."

The disease of the kidneys and the adhesions between the pleuræ the writer regards as intercurrent affections, and not necessarily connected with the disease of the lungs. He very correctly shows the error of Dr. Holland's notions concerning the state of the bronchial glands and dilatation of the bronchi as constituting peculiar phenomena in this disease.

The primary or essential changes are inflammation of the larynx, trachea, and bronchi, small bodies resembling currants, and tubercles. The laryngeal and tracheal inflammation, with occasional ulceration, is said by Dr. Holland to be in a large number of cases the primary affection—he holds that it remains sometimes for a considerable period. This Dr. Favell "doubts." But Dr. Holland says, that on examining "the larynx during the continuance of these symptoms, the mucous membrane is often much more florid and vascular than is natural, and occasionally small ulcerated points are observed." And now behold Dr. Favell has Dr. Holland on the hip, and, having him there, is somewhat merciless towards his colleague and townsman: "Now, this statement I shall not attempt to contravene, inasmuch as I do not pretend to be able to inspect the lining membrane of the larynx during the lifetime of the patient." Dr. Favell is evidently sharper than Dr. Holland; his sidelong cuts at the rival historian are remarkable enough. Indeed, if we may judge from Dr. Favell's exhibition of temper, it would seem probable that the existence of harmony scarcely prevails amid the doctors of Sheffield; and we cannot but think it would have been more dignified and decorous in the author to have shown a less critical spirit in dealing with the opinions of his townsman, more especially in a paper publicly read in an assembly of their *local brethren.*

The "currant-like bodies" Dr. Favell considers are nothing more than the dilated extremities of veins, containing some of the solid constituents of the blood. He says that he has traced such appearances with the scalpel. Were we disposed to be critical, we might fairly question Dr. Favell's being any more able to trace the *extremities* of veins with the scalpel, than Dr. Holland to see the interior of the larynx in the living man; but we understand both, and we merely advert to the circumstance from the natural suggestion of stones and glass houses.

Dark masses as big as a nut or as an orange—sometimes grayish, sometimes black—sometimes dense, sometimes easily cut, are found in some cases. What are these? "In some instances I believe they are occasioned by the effusion of blood into the parenchymatous substance of the lungs, constituting what is properly called pulmonary apoplexy; but in the majority of cases they are doubtless the consequence of pneumonia, either in the acute or chronic form, and to which the grinders are peculiarly liable." Now this "sometimes one, and sometimes the other" mode of getting rid of a difficulty, reminds us of the well-known answer of the Cantab. at the "Little-Go," who to the question of his good-natured examiner, as to whether the sun moved round the earth, or the earth round the sun, boldly replied (by way of making sure) "sometimes one and sometimes the other." Dr. Favell must surely have felt that as two states could never be more dissimilar in nature and pathological signification than hemorrhage and inflammation,—yet he makes them have almost convertible morbid changes.

On the whole this essay gives us a rather more precise account of the errors and inadvertences of Dr. Holland than of the real nature of the disease, concerning which it professes to enlighten us. It, however, exhibits talent; and we are glad to receive it as an earnest of something better on the same subject from the same pen. The author's general conclusion is that "the disease essentially depends on congestion, or inflammation of the parenchymatous structure of the lungs; in some cases giving rise to the formation of tubercle, and in others occasioning pulmonary degeneration without tubercular deposit."

---

